# Relative faecal abundance to predict extended-spectrum β-lactamase-producing *Enterobacterales* related ventilator‑associated pneumonia

**DOI:** 10.1186/s13613-025-01456-w

**Published:** 2025-03-20

**Authors:** Pierre Bay, Paul-Louis Woerther, Vincent Fihman, Ségolène Gendreau, Pascale Labedade, Antoine Gaillet, Florian Jolly, Guillaume Carteaux, Nicolas de Prost, Jean-Winoc Decousser, Armand Mekontso-Dessap, Keyvan Razazi

**Affiliations:** 1 DMU Médecine, Service de Médecine Intensive Réanimation, AP-HP (Assistance Publique-Hôpitaux de Paris), Hôpitaux Universitaires Henri Mondor, CHU Henri Mondor, 51, Av. de Lattre de Tassigny, CEDEX, 94010 Créteil, France; 2 Faculté de Santé de Créteil, UPEC (Université Paris Est Créteil), IMRB, GRC CARMAS, 94010 Créteil, France; 3 UPEC (Université Paris Est), INSERM, Unité U955, Équipe 18, 94010 Créteil, France; 4 Département de Virologie, Bactériologie, Parasitologie-Mycologie, AP-HP (Assistance Publique-Hôpitaux de Paris), Hôpitaux Universitaires Henri Mondor, 94010 Créteil, France; 5 UPEC (Université Paris Est), EA 7380 Dynamic, Ecole Nationale Vétérinaire d’Alfort, USC Anses, Créteil, France

## Abstract

**Background:**

Antimicrobial stewardship (AMS) for ventilator-associated pneumonia (VAP) in carriers of extended-spectrum β-lactamase-producing *Enterobacterales* (ESBL-E) presents significant challenges. The abundance of ESBL-E rectal carriage has emerged as a potentially valuable tool for predicting ESBL-E-related VAP.

**Methods:**

This single-center, retrospective study was conducted between October 2019 and April 2023 in the medical ICU of a university hospital. The relative abundance of ESBL-E rectal carriage (RAC) was calculated as the ratio of ESBL-E counts to the total number of aerotolerant bacteria. The aim was to evaluate the predictive value of RAC for diagnosing ESBL-E-related VAP in patients with confirmed VAP who were ESBL-E carriers.

**Results:**

During the study period, 478 patients with ESBL-E carriage were admitted to the ICU, of whom 231 (48%) required mechanical ventilation. Eighty-three patients (17%) developed a total of 131 confirmed VAP episodes, of which 62 episodes (47%) were ESBL-E-related VAP. The median interval between the last rectal screening and VAP occurrence was 4 [3–7] days. RAC was not associated with ESBL-E-related VAP in the entire cohort (p = 0.39). Similar findings were observed in several sensitivity analyses, including the following subsets: recent and high-quality screening (interval between screening and VAP ≤ 7 days and bacterial load on rectal swab > 10^4^ CFU/mL, p = 0.21); first VAP episodes only (p = 0.41); cases involving *Escherichia coli* exclusively (p = 0.08) or other ESBL-E strains (p = 0.29); and VAP associated with Gram-negative bacteria (p = 0.26) or *Enterobacterales *(p = 0.34). However, in a multivariable model, rectal colonization with non-*Escherichia coli* ESBL strains was independently associated with ESBL-E-related VAP (adjusted odds ratio [aOR] 1.213 [95% CI 1.005–1.463], p = 0.045).

**Conclusion:**

RAC was not associated with confirmed VAP in ESBL-E carriers. Further studies are needed to explore effective strategies for improving AMS in ESBL-E carriers with suspected VAP.

**Supplementary Information:**

The online version contains supplementary material available at 10.1186/s13613-025-01456-w.

## Introduction

Rational antimicrobial stewardship (AMS) in the intensive care unit (ICU) requires balancing the need for an early and adequate antibiotic regimen against the risk of promoting multidrug-resistant (MDR) bacteria through unnecessary broad-spectrum antibiotic use [1]. AMS poses particular challenges in patients colonized with extended-spectrum β-lactamase-producing *Enterobacterales *(ESBL-E), a known risk factor for ESBL-E-related infections. Ventilator-associated pneumonia (VAP) is the most common ICU-acquired infection [2]. French guidelines recommend carbapenem use for suspected VAP in ESBL-E-colonized patients who are immunosuppressed or exhibit severe infection [3]. However, ESBL-E-related VAP accounts for only 7% of infection-related ventilator-associated complications in carriers [4]. Importantly, ESBL-E rectal carriage is a well-recognized driver of carbapenem overuse in the ICU [5, 6], emphasizing the need for novel diagnostic strategies in ESBL-E carriers with suspected VAP.

Several studies have demonstrated that intestinal colonization with ESBL-E is a prerequisite for the development of ESBL-E infections [7, 8]. Research that defines the relative abundance of ESBL-E as the ratio of ESBL *Enterobacterales *to the total *Enterobacterales *count in stool samples suggests that the relative or absolute abundance of ESBL-E in the gut may enhance risk assessment for ESBL infections in carriers [9, 10]. The hypothesis of this study was that the relative abundance of ESBL-E rectal carriage (RAC), calculated as the ratio of ESBL-E counts to the total number of aerotolerant bacteria, on the last rectal swab prior to the onset of VAP would be associated with the ESBL-E status of VAP and could serve as an effective tool to guide empiric antibiotic therapy in cases of suspected VAP in ESBL-E carriers.

The primary aim of this study was to evaluate the performance of RAC in diagnosing confirmed ESBL-E-related VAP in ESBL-E carriers. The secondary aim was to identify factors associated with ESBL-E-related VAP in ESBL-E carriers.

## Methods

### Settings and patients

This single-center, retrospective study was conducted between October 2019 and April 2023 in the medical ICU of a university hospital that did not used selective digestive decontamination (SDD). All confirmed VAP episodes in ESBL-E carriers were included. Data collected included age, sex, comorbidities, Simplified Acute Physiology Score (SAPS II), primary reason for admission, antibiotic classes administered during the ICU stay, clinical and biological factors at the time of respiratory sampling, and characteristics of ESBL-E carriage.

### Definitions

Confirmed VAP was defined as clinical suspicion of VAP combined with a positive quantitative culture from a respiratory sample. VAP was clinically suspected after 48 h of mechanical ventilation in the presence of new or persistent pulmonary infiltrates on chest X-ray, along with at least two of the following classical signs: purulent tracheal secretions, fever or hypothermia (body temperature ≥ 38.5 °C or ≤ 36.5 °C), leukocytosis or leukopenia (white blood cell count ≥ 12 × 10⁹/L or ≤ 4 × 10⁹/L) [4, 11, 12]. VAP confirmation required a quantitative culture [12] from one of the following samples: protected telescopic catheter (≥ 10^3^ CFU/mL), bronchoalveolar lavage fluid (≥ 10^4^ CFU/mL), or endotracheal aspirate (≥ 10^5^ CFU/mL).

### Microbiological analysis

Analysis of respiratory samples included standardized quantitative culture as previously described [13] and bacterial identification using Matrix-Assisted Laser Desorption/Ionization-Time-Of-Flight (MALDI-TOF) mass spectrometry (Microflex LT, Bruker Daltonics, Bremen, Germany). Bacterial species present in quantities exceeding the defined thresholds for the respective sample types were considered involved in the infection process. Antibiotic susceptibility testing was performed using the disc diffusion method on Mueller–Hinton media (Bio-Rad, Marnes-la-Coquette, France) in accordance with EUCAST recommendations (https://www.eucast.org/ast_of_bacteria). For Enterobacterales, ESBLs were phenotypically detected during susceptibility testing if a difference of more than 5 mm was observed between the *cefepime* and *cefepime* + *clavulanate* discs and/or using a double-disc synergy test [14].

In our hospital, RAC was routinely assessed using an automated procedure [15]. Rectal swabs were collected from each patient within 24 h of ICU admission as part of standard care and then weekly throughout the ICU stay. Stool samples were collected using a transport medium (FecalSwab®, Copan Diagnostics, Brescia, Italy). 30 µL of the Amies liquid contained in the FecalSwab® was plated by an automated inoculator (WASP®, Copan Diagnostics) onto a non-selective Tryptone Soya Agar medium (TSA, Oxoid, Wesel, Germany) and a selective CHROMID® ESBL medium (bioMérieux, Marcy l’Étoile, France). Plates were incubated for 24–36 h at 35 °C under aerobic conditions. For colonies grown on the ESBL medium, ESBL production was confirmed using the β-LACTA™ test (BioRad, Marnes-la-Coquette, France). If results were inconclusive, ESBL production was confirmed through susceptibility testing.

**Fig. 1:**
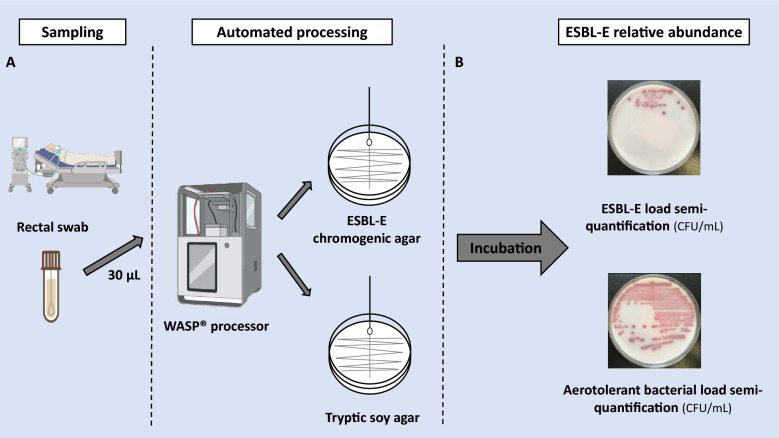
Relative abundance of ESBL-E rectal assessment. **A** Automated seeding of rectal swabs using WASP® instrument (PREVI Isola, bioMérieux, France) on chromogenic agar (Oxoid Ltd, Cambridge, UK; Biomérieux, Courtaboeuf, France) for ESBL-E semi-quantification assessment and on Tryptic soy agar for aerotolerant bacterial. **B** Semi-quantitative counting of bacteria after incubation

After incubation, the aerotolerant bacterial load on the swab was semi-quantified by colony counting on TSA as previously described [13, 15]. Bacterial loads were classified as: negative (< 10^3^ colony-forming units (CFU)/mL); very low (≤ 10^3^ CFU/mL); low (> 10^3^ CFU/mL and ≤ 10^4^ CFU/mL); moderate (> 10^4^ CFU/mL and ≤ 10^5^ CFU/mL); high (> 10^5^ CFU/mL and ≤ 10^6^ CFU/mL); and very high (> 10^6^ CFU/mL). The relative abundance of ESBL-E (RAC) was calculated as the ratio of ESBL-E counts to the total number of aerotolerant bacteria, expressed as a percentage (Fig. 1). For patients carrying multiple ESBL-E strains, the RAC of the dominant clone was used.

As previously reported, the risk of ESBL-E-related VAP varies by ESBL-E species [16]. Therefore, subgroup analyses were performed, distinguishing rectal colonization with *Escherichia coli* alone from other *Enterobacterales *species.

### Statistical analysis

Descriptive results are presented as means (± standard deviation) or medians (1st–3rd quartiles) for continuous variables, and as numbers with percentages for categorical variables. Unadjusted comparisons based on ESBL-E-related VAP status (ESBL-E-related vs. non-ESBL-E-related) were conducted using Chi-square or Fisher’s exact tests for categorical variables, and t-tests or Mann–Whitney U tests for continuous variables, as appropriate. Cochran-Armitage trend tests were employed to compare RAC results between episodes with and without ESBL-E-related VAP. Sensitivity analyses were performed in the following subpopulations: (i) VAP episodes with recent and high-quality screening (i.e., a time interval between VAP occurrence and the last rectal screening ≤ 7 days and a bacterial load on rectal swabs > 10^4^ CFU/mL) [14]; (ii) only the first episode of VAP; (iii) based on ESBL-E colonization type (*Escherichia coli* only vs. other ESBL-E); (iv) VAP episodes caused by Gram-negative bacteria; and (v) VAP episodes caused by Enterobacterales.

To identify independent factors associated with ESBL-E-related VAP, we analyzed non-redundant variables that were associated in univariate analysis (p < 0.01) and deemed clinically relevant using backward logistic regression. The clinically relevant variables selected a priori included: RAC value from the most recent rectal screening prior to the occurrence of VAP, SOFA score on the day of VAP [17]; species of ESBL *Enterobacterales *in rectal colonization [16]; time between intubation and VAP [17]; and carbapenem use within 72 h prior to sampling [4]. The associated factors at the time of VAP were the primary variables of interest for predicting ESBL-E-related VAP. To prevent overfitting, we limited each model to a maximum of five variables, ensuring a minimum of five outcome events per predictor variable [18].

Statistical significance was defined as a p < 0.05. Analyses were conducted using IBM SPSS Statistics software (version 22.0, IBM Corp, Armonk, NY) and RStudio software (version 4.2.0, https://www.r-project.org/). The methods and results of this study are reported in accordance with the STROBE guidelines [19].

### Ethical considerations

This observational study was approved by the Ethics Committee of the French Intensive Care Society (CE SRLF 24-009, IRB N°00014135). Patients were informed about their inclusion in the study, and written consent was waived in accordance with French law. The database is registered with the “Commission Nationale de l’Informatique et des Libertés” (n°2232944).

## Results

### Population

**Fig. 2:**
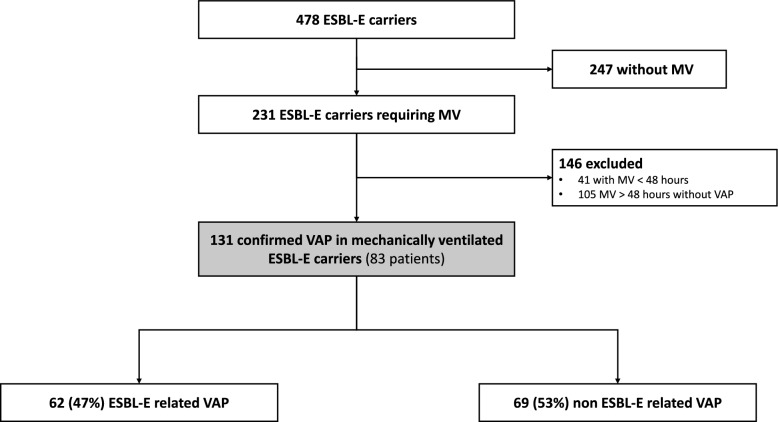
Flow chart of the 131 confirmed VAP episodes in mechanically ventilated ESBL-E carriers. ESBL-E: extended-spectrum β-lactamase-producing *Enterobacterales*; MV: mechanical ventilation; VAP: ventilator associated pneumonia

During the study period, 478 patients were identified as ESBL-E rectal carriers. Among them, 231 (48.3%) required invasive mechanical ventilation (Fig. 2). Of these carriers, 83 patients developed 131 episodes of VAP (Fig. 2), including 62 episodes (47%) of ESBL-E-related pneumonia. Among the 83 carriers who developed VAP, 50 patients (60.2%) had ESBL-E carriage detected on admission screening, primarily with *Escherichia coli* only (N = 35/50, 70%). The remaining 33 patients (40%) acquired ESBL-E carriage during their ICU stay, predominantly with *Enterobacter cloacae* or *Klebsiella pneumoniae* (N = 19/33, 58%). The evolution of RAC in consecutive rectal swabs varied throughout the ICU stay (eFigure 1). Patient characteristics are summarized in Table 1. Two-thirds of the patients were admitted to the ICU for acute respiratory failure, and half for severe COVID-19 pneumonia.

**Table 1:** Patient’s characteristics (*N* = 83) at ICU admission and during ICU stay

Variable	Patients, *N* = 83
Age, years	61 [51–68]
Sex, females	26 (31)
Body mass index, kg/m^2^	27.4 [24.1–32]
*Comorbidities*	
Charlson Comorbidity index	1 [0–3]
Hypertension	46 (55)
Diabetes mellitus	28 (34)
Congestive heart failure (NYHA 3–4)	7 (8)
COPD	7 (8)
Immunosuppression condition	13 (16)
SAPS II at ICU admission, points	49 [33–66]
*Reason for ICU admission*	
Shock	15 (18)
Acute respiratory failure	56 (67)
Coma	5 (6)
Cardiac arrest	7 (8)
COVID-19 related admission	40 (48)
*In-ICU management*	
Vasopressor support	75 (90)
Prone positioning	50 (60)
Renal Replacement Therapy	35 (42)
ECMO	22 (26)
In-ICU mortality	30 (36)

Results are *N* (%), means (± standard deviation) or medians (interquartile range, i.e., quartile 1; quartile 3)

COVID-19: Coronavirus Disease 2019; ESBL-E: extended-spectrum β-lactamase-producing *Enterobacterales;* ECMO: extracorporeal membrane oxygenation; ICU: Intensive Care Unit; NYHA: New York Heart association; SAPS: simplified acute physiology score; VAP: ventilator-associated pneumonia

### ESBL-E related VAP characteristics

Results of conventional microbiology are presented in Table 2. Among the 62 episodes of ESBL-E-related VAP, the most frequent species identified were *Klebsiella pneumoniae* (N = 22, 36%), *Escherichia coli* (N = 21, 34%), *Enterobacter cloacae* (N = 19, 31%), and *Klebsiella oxytoca* (N = 1, 2%). In most cases of ESBL-E-related VAP (N = 59/62, 95.2%), the species matched those found in rectal carriage.

**Table 2:** Results of microbiology of the 131 episodes of VAP in mechanically ventilated ESBL-E carriers

Variable	ESBL-E related VAP group, n = 62	Non ESBL-E related VAP, n = 69
Enterobacterales		
Non-ESBL *Enterobacterales *VAP	n.a	32 (46)
* Escherichia coli*	22 (35)	4 (6)
* Klebsiella pneumoniae*	25 (40)	9 (13)
* Enterobacter cloacae*	22 (35)	2 (3)
* Klebsiella aerogenes*	4 (6)	4 (6)
* Klebsiella oxytoca*	0	2 (3)
* Proteus mirabillis*	2 (3)	3 (4)
* Serratia marcescens*	3 (5)	0
* Hafnia alvei*	0	1 (1)
* Citrobacter koseri*	0	1 (1)
* Raoultella ornithinolytica*	0	1 (1)
EBSL-E related pneumonia		
* Escherichia coli*	21 (34)	0
* Klebsiella pneumoniae*	22 (35)	0
* Enterobacter cloacae*	19 (31)	0
Nonfermenting gram-negative bacilli		
* Pseudomonas aeruginosa*	18 (29)	21 (30)
* Stenotrophomonas maltophilia*	7 (11)	12 (17)
* Acinetobacter *spp.	2 (3)	4 (6)
* Achromobacter xylosoxidans*	4 (6)	1 (1)
* Burkholderia *spp.	0	2 (3)
* Haemophilus influenzae*	0	1 (1)
* Prevotella *spp.	0	1 (1)
* Pantoea *spp.	0	1 (1)
Gram positive bacteria		
* Staphylococcus aureus*	2 (3)	10 (14)
* Streptococcus *spp.	1 (2)	4 (6)
* Corynebacterium striatum*	1 (2)	4 (6)
* Staphylococcus haemolyticus*	0	1 (1)
* Enterococcus faecium*	0	1 (1)
Carbapenem-resistant bacteria	10 (16)	24 (35)
Polymicrobial VAP	37 (60)	27 (39)

ESBL-E: extended-spectrum β-lactamase-producing *Enterobacterales;* VAP: ventilator-associated pneumonia

Among the 69 episodes of non-ESBL-E-related VAP, 32 episodes (46%) were associated with Enterobacterales. The most commonly identified bacteria were *Pseudomonas aeruginosa* (N = 21, 31%), *Stenotrophomonas maltophilia* (N = 12, 17%), and *Staphylococcus aureus* (N = 10, 15%). Thirty-four VAP episodes (26%) were caused by carbapenem-resistant bacteria (CRB).

**Fig. 3:**
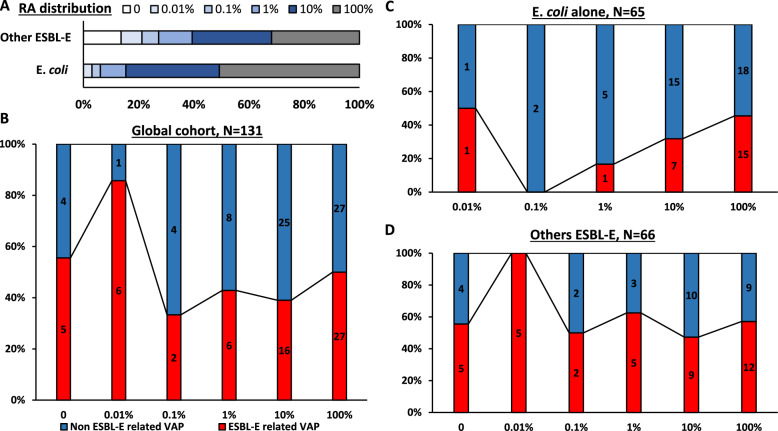
Relative abundance of ESBL-E rectal carriage (RAC) according to ESBL-E related VAP status. **A** RAC distribution according to ESBL-E colonisation (*Escherichia coli* only vs. other ESBL-E). **B** RAC according to ESBL-E related VAP status of the 131 confirmed ventilator-associated pneumonia. **C** RAC according to ESBL-E related VAP status of the 65 confirmed ventilator-associated pneumonia in *Escherichia coli* carriers. **D** RAC according to ESBL-E related VAP status of the 66 confirmed ventilator-associated pneumonia in non *Escherichia coli* carriers. ESBL-E: extended-spectrum β-lactamase-producing *Enterobacterales*; RAC: relative abundance of ESBL-E rectal carriage; VAP: ventilator-associated pneumonia. P-values come from unadjusted comparisons using Cochran–armitage trend test

Characteristics of ESBL-E-related and non-ESBL-E-related VAP are reported in Table 3. Data on empiric and curative antibiotic therapy according to ESBL-E-related VAP are detailed in Table 3 and eTable 1, respectively. The distribution of RAC categories (Fig. 3) based on the last rectal screening prior to VAP occurrence showed no significant differences between ESBL-E-related VAP and non-ESBL-related VAP (p = 0.20, eTable 2). Similarly, the absolute semi-quantification of ESBL-E bacterial load did not differ significantly by ESBL-E-related VAP status (p = 0.48, eTable 2).

**Table 3:** Characteristics of the 131 confirmed VAP according to ESBL-E related VAP status

Variable	ESBL-E related VAP, n = 62	Non ESBL-E related VAP, n = 69	p
Days after admission	24 [12–46]	12 [8–26]	**0.006**
Duration of MV before VAP	21 [11–39]	11 [6–25]	**0.006**
Previous VAP	41 (66)	26 (38)	**0.001**
Number of previous VAP	2 [1–3]	2 [1–4]	0.47
*Patient characteristics on the day of the sample*			
Antibiotics received within 72 h prior to sample	39 (63)	40 (58)	0.56
Carbapenem received within 72 h prior to sample	10 (16)	9 (13)	0.62
ECMO	19 (31)	11 (16)	**0.046**
SOFA score	7 [4–11]	7 [4–11]	0.69
PaO_2_/FiO_2_	156 [81–260]	160 [93–240]	0.92
PaO_2_/FiO_2_ < 150 mmHg	29 (47)	29 (42)	0.58
Circulatory failure^a^	29 (47)	34 (49)	0.77
Antibiotic therapy on the day of the sample	27 (44)	28 (41)	0.73
Carbapenem on the day of the sample	8 (13)	3 (4)	0.08
ESBL-E colonisation			
* ESBL-E* species			**0.007**
* Escherichia coli* alone	24 (39)	41 (59)	
* K. pneumoniae* and/or *E. cloacae*	37 (60)	23 (33)	
Others^b^	1 (2)	5 (7)	
In-ICU acquired ESBL-E carriage	41 (66)	26 (38)	**0.001**
Empiric antibiotherapy			
No initiation	19 (31)	24 (35)	0.61
Non-carbapenem β-lactam	19 (31)	23 (33)	0.74
Carbapenem	24 (39)	22 (32)	0.41
Adequate empiric antibiotic therapy^c^	28 (45)	36 (52)	0.42

Results are *N* (%), means (± standard deviation) or medians (interquartile range, i.e., quartile 1; quartile 3)

CFU: colony forming unit; ECMO extracorporeal membrane oxygenation; ESBL-E: extended-spectrum β-lactamase-producing *Enterobacterales*; MV: mechanical ventilation; PaO_2_/FiO_2_: ratio of the partial pressure of arterial oxygen to the fraction of inspired oxygen; SOFA: sequential organ failure assessment; VAP: ventilator associated pneumonia

^a^Circulatory failure is defined as cardiovascular SOFA score ≥ 3

^b^*Klebsiella Oxytoca* (n = 2), *Citrobacter Amalonaticus* (n = 1), *Klebsiella Aerogenes* (n = 1), *Citrobacter Koseri* (n = 1), *Citrobacter Freundii* (n = 1)

^c^Empiric antibiotic therapy was considered as optimal if it was not only active but also not excessively broad-spectrum

Two-tailed *p*-values come from unadjusted comparisons using Chi-square or Fisher’s exact tests for categorical variables, and *t*-tests or Mann–Whitney tests for continuous variables, as appropriate. No adjustment for multiple comparisons was performed. Bolded p-values are significant at the p < 0.05 level

Sensitivity analyses yielded similar results (eTable 2), including subsets limited to: recent and high-quality screening (N = 85, p = 0.10), only the first episode of VAP (N = 83, p = 0.45), patients with rectal carriage of *Escherichia coli* only (N = 65, p = 0.10, Fig. 3C) or other ESBL-E strains (N = 66, p = 0.22, Fig. 3D), episodes related to at least one Gram-negative bacterium (N = 120, p = 0.15), or episodes caused by *Enterobacterales *(N = 94, p = 0.23).

The diagnostic performance of RAC for ESBL-E-related VAP was poor, with a negative predictive value ≤ 80% regardless of the threshold used or the rectal carriage strain (*Escherichia coli* vs. others) (eTable 3).

### Factors associated with ESBL-E-related VAP

Acquired ESBL-E colonization during the ICU stay and rectal carriage with non-*Escherichia coli* ESBL *Enterobacterales *were significantly associated with ESBL-E-related VAP (Table 3). The duration of mechanical ventilation before VAP was longer in patients with ESBL-E-related VAP compared to those with non-ESBL-E-related VAP (21 [11–39] vs. 11 [6–25] days, p = 0.009). Additionally, patients with ESBL-E-related VAP were more likely to have had a previous episode of VAP than those with non-ESBL-E-related VAP (66.1% (N = 41/62) vs. 37.7% (N = 26/69), p = 0.001).

Except for extracorporeal membrane oxygenation support, other indicators of severity on the day of VAP—such as SOFA score, PaO_2_/FiO_2_ ratio, and circulatory failure—did not differ significantly between ESBL-E-related and non-ESBL-E-related VAP cases. Both groups had similar exposure to antibiotic therapy within 72 h before the VAP episode.

In the multivariable analysis (Table 4), non-*Escherichia coli* ESBL *Enterobacterales *carriage was independently associated with ESBL-E-related VAP (aOR 1.213 [95% CI 1.005–1.463], p = 0.045). However, semi-quantitative RAC categories of ESBL-E rectal colonization were not significantly associated with ESBL-E-related VAP.

**Table 4:** Factors associated with the occurrence of ESBL-E related VAP by multivariable logistic regression models in ESBL-E carriers with confirmed VAP (*N* = 131)

Factor	Univariable analysis			Multivariable analysis		
	OR	95%CI	p-value	aOR	95%CI	p-value
Relative abundance of ESBL-E rectal colonisation (semi-quantitative)^a^						
0 (ref)						
0.01%	4.8	[0.497–111.687]	0.2	1.35	[0.823–2.203]	0.24
0.1%	0.4	[0.0395–3.25]	0.4	0.89	[0.527–1.498]	0.66
1%	0.6	[0.105–3.24]	0.6	0.95	[0.624–1.433]	0.79
10%	0.51	[0.112–2.212]	0.4	0.93	[0.641–1.336]	0.68
100%	0.8	[0.181–3.34]	0.8	1.06	[0.737–1.524]	0.75
In-ICU acquired ESBL-E carriage^b^	3.01	[1.489–6.216]	0.002		Not included	
ESBL *Enterobacterales *rectal colonisation						
* Escherichia coli* alone (ref)						
Non-*Escherichia coli* alone strains	2.32	[1.157–4.725]	0.02	1.21	[1.003–1.455]	0.049
Time between intubation and VAP	1.01	[1.000–1.017]	0.08	1	[0.999–1.003]	0.51
SOFA score on the day of the sample	0.99	[0.905–1.076]	0.8	1	[0.975–1.019]	0.77
Carbapenem received within 72 h prior to sample	1.28	[0.481–3.46]	0.62	0.89	[0.682–1.149]	0.36
Previous VAP	2.66	[1.289–5.607]	0.01	1.2	[0.978–1.465]	0.08

The multivariable model showed a good calibration as assessed by the Hosmer and Lemeshow goodness-of-fit test (χ^2^ = 5.0516, p = 0.752) and an acceptable discrimination as assessed by the receiver operating characteristics curve (area under the curve = 0.7008)

aOR (CI 95%): adjusted Odds Ratio (95% confidence interval); ESBL-E: extended-spectrum β-lactamase-producing *Enterobacterales*; ICU: intensive care unit; SOFA: sequential organ failure assessment; VAP: ventilator associated pneumonia

p-values come from multivariable logistic regression models

^a^RAC on the last rectal screening available at the occurrence of VAP

^b^Not included as 75% of acquired ESBL were non-*Escherichia coli* alone strains. These variables were considered associated, and only ESBL *Enterobacterales *rectal colonisation was considered for multivariable analysis

## Discussion

This retrospective study of 131 VAP episodes in ESBL-E carriers found that routine RAC of ESBL-E in rectal swabs, calculated using the total aerotolerant bacterial count as the denominator, was not associated with ESBL-E-related VAP. In multivariable analysis, rectal colonization with non-*Escherichia coli* ESBL *Enterobacterales *was associated with ESBL-E-related VAP. These findings suggest that RAC alone may not be sufficient to guide empiric antibiotic therapy in ESBL-E carriers with suspected VAP.

The main strength of this study lies in its evaluation of RAC using a pragmatic and reproducible approach. Given that approximately two-thirds of ICUs globally screen for MDR carriage upon admission and/or during the ICU stay [20], this method has the potential for broad applicability. In our study, RAC was assessed through rectal swabs routinely collected at ICU admission and then weekly, minimizing additional laboratory or nursing workload. Furthermore, an automated assessment of RAC of ESBL-E was used, a tool that is routinely available to clinicians, adding practical value in real-world ICU settings.

AMS in ESBL-E carriers remains a significant challenge for intensivists. For carriers with severe disease, current guidelines recommend carbapenems as empiric therapy for suspected VAP [3]. However, the benefit of detecting intestinal ESBL-E carriage through active surveillance cultures for carbapenem stewardship remains debated [6]. While some studies suggest that this approach improves the likelihood of adequate empiric antibiotic therapy [20], it may also contribute to carbapenem overuse [5]. Although the negative predictive value of rectal screening for ESBL-E colonization is high (> 90%), it is not absolute [7, 21]. False negatives [15] and the potential acquisition of carriage between the last available rectal screening and the onset of infection contribute to this limitation. Moreover, a negative rectal screening cannot exclude infections caused by other Gram-negative bacteria requiring carbapenem therapy, such as ceftazidime-resistant *Pseudomonas aeruginosa* or MDR *Acinetobacter baumannii*. On the other hand, the low positive predictive value of rectal screening for predicting ESBL-E-related infections in carriers, combined with the absence of robust individual predictive factors, often results in the overuse of carbapenems in colonized patients. Consequently, such surveillance cultures might only be justified in ICUs with a relatively high prevalence of ESBL-E colonization (e.g., > 5–10%) and a restrictive antibiotic policy [5, 6, 22, 23].

Our study demonstrates that generalizing carbapenem use for all ESBL-E carriers is not an appropriate strategy. First, as previously observed, a quarter of VAP cases in our cohort were caused by CRB [16]. Second, unnecessary exposure to carbapenems increases the risk of inducing CRB in future infections [24–26]. Third, recent studies have highlighted the positive impact of restrictive antibiotic policies on reducing MDR bacterial emergence [27, 28]. These factors strongly advocate for the validation of novel diagnostic approaches (e.g., multiplex rapid PCR, RAC).

The integration of RAC has been proposed as a promising tool to guide empiric antibiotic therapy in ESBL-E carriers across different contexts: (i) in a study of ESBL *Escherichia coli* urinary tract infections (UTIs) in women, fecal ESBL-RAC was found to correlate with the occurrence of ESBL-E UTIs, with an ESBL-RAC of < 0.1% being 100% predictive of a non-ESBL *E. coli* UTI [9]; (ii) a pilot study of 24 predominantly ESBL-producing *E. coli* carriers who developed infections during ICU stay found that fecal ESBL-RAC was associated with ESBL-E-related infections [10]; (iii) a study found an association between the level of ESBL-E intestinal carriage (semi-quantitative assessment in stool samples) and the risk of ESBL-E related bloodstream infections in patients with haematological malignancies [29]; (iv) and another monocentric study focusing on VAP showed that a high-density of ESBL-E rectal carriage is a risk factor for ESBL-E VAP in case of GNB-related VAP [30].

Our findings differ from these studies, likely due to methodological differences. These studies are summarized in eTable 4. The relative abundance of ESBL-E in studies by Ruppé et al. and Pilmis et al. was calculated from stool samples as the ratio of ESBL *E. coli* counts to the total number of *Enterobacterales *(determined by plating serial dilutions), expressed as a percentage [9, 10]. In contrast, studies by Woerther et al. and Andremont et al. assessed absolute abundance of ESBL-E using semi-quantitative methods—either through stool cultures [29] or rectal swab cultures [30]. Using stool samples instead of rectal swabs for RAC calculation might yield more reliable results. However, this approach is challenging in critically ill patients, who often have impaired intestinal transit [31]. Furthermore, ICU patients—particularly ESBL-E carriers—frequently exhibit significant disruption of the normal intestinal microbiota, which is primarily composed of resident anaerobes [32, 33]. Therefore, using aerotolerant bacteria as the denominator for RAC calculation instead of *Enterobacterales *may be more accurate, as these bacteria represent the most common pathogens in healthcare-associated infections [34]. In addition, using absolute ESBL-E abundance through semi-quantitative culture rather than RAC may be influenced by sampling fluctuations, as absolute abundance is affected by the quality of rectal swabs [15], making it less reliable.

The lack of association between RAC and ESBL-E-related VAP in our study raises questions about the relationship between gastrointestinal and orotracheal colonization in critically ill patients on mechanical ventilation. Although several studies have demonstrated a correlation between MDR colonization at these two sites [30, 35, 36], predicting MDR-related VAP may be more accurate when monitoring lower respiratory tract colonization [30, 37–39]. However, approaches focusing on respiratory tract colonization (oropharyngeal or tracheal) are less pragmatic than those based on rectal colonization, may lead to antibiotic overuse, and require further prospective studies to assess their impact.

Other diagnostic tools could improve AMS in this context, such as rapid microbiological diagnostics using multiplex PCR (mPCR) platforms like BioFire FilmArray® or Unyvero®. For instance, mPCR targeting blaCTX-M has demonstrated excellent diagnostic accuracy in ruling out ESBL-E-related pneumonia in ESBL-E carriers with suspected VAP or vHAP [40]. However, in cases with a very low pre-test probability of pneumonia, the high sensitivity of these tools may lead to overdiagnosis and carbapenem overuse. Additionally, their cost-effectiveness and prognostic impact remain uncertain [41], especially in populations at high risk of MDR infections with complex resistance mechanisms that cannot be fully predicted until definitive culture and antimicrobial susceptibility results are available [42].

Consistent with previous studies [16, 30, 43, 44], our findings confirm that non-*Escherichia coli* ESBL *Enterobacterales *carriage is a predictive factor for ESBL-E-related VAP. Several factors may explain the association between non-*Escherichia coli* carriage and the risk of ESBL-E-related VAP. First, studies have shown that environmental contamination with ESBL-E *Klebsiella pneumoniae* is higher than with ESBL-E *Escherichia coli* [45, 46]. This increased environmental presence may raise the likelihood of respiratory colonization and subsequent infection in ventilated patients. Second, infections caused by *Klebsiella pneumoniae* are often associated with greater severity compared to those caused by *Escherichia coli* [47, 48], which could lead to more frequent diagnoses. Third, certain *Klebsiella pneumoniae* strains possess an enhanced ability to form biofilms [49], a factor that may facilitate colonization and persistence in the respiratory tract, even under antimicrobial pressure.

Further studies investigating the genomic characteristics, virulence factors, and environmental contamination potential of non-*Escherichia coli* ESBL-producing strains are warranted. Such research could help clarify the association between non-*Escherichia coli* carriage and the elevated risk of ESBL-E-related VAP, providing insights to refine infection control measures and AMS strategies for ESBL-E carriers in ICUs.

Interestingly, in our study, the duration of mechanical ventilation before VAP was significantly longer in the ESBL-E-related VAP group. However, this factor was not independently associated in the multivariable analysis. Although other studies have reported this association [7], this finding could also reflect the effect of acquiring ESBL-E carriage during the ICU stay on the risk of developing ESBL-E infection, as recently observed [50].

Our study has several limitations. First, it is a monocentric and retrospective analysis, which limits the ability to infer causality. Second, we included all VAP episodes regardless of microbiology (i.e., not exclusively Enterobacterales-related episodes). While this approach may introduce heterogeneity, it reflects a more pragmatic, “real-world” use of this tool. Importantly, sensitivity analyses focusing on gram-negative bacteria-related VAP or Enterobacterales-related VAP yielded consistent results, supporting the robustness of our findings. Third, the quality and timing of rectal sampling are critical factors to consider. As previously demonstrated, poor-quality rectal swabs (e.g., those with low bacterial loads) are associated with underdetection of ESBL-E [15], potentially influencing the results. Future studies should aim to standardize rectal sampling protocols to ensure more reliable data collection. It is also reasonable to hypothesize that rectal sampling performed long before the onset of VAP may have limited ability to identify the pathogen responsible for the VAP. Nevertheless, our study specifically investigated RAC, which reduces the impact of poor-quality sampling. Moreover, the results remained consistent in the sensitivity analysis that included only VAP episodes with a rectal swab sampled within the preceding 7 days and a high bacterial load (i.e., > 10^4^ CFU/mL) [14]. Notably, the median interval between VAP onset and the last available rectal swab was 4 days, which aligns with previous studies [30]. Fourth, ESBL-RAC was calculated using aerotolerant bacteria rather than the entire gut microbiota. In addition, unlike previous studies that used *Enterobacterales *as the denominator, we used all aerotolerant bacteria in the RAC calculation. This methodological choice may have influenced our findings, as the relative proportion of ESBL-E among aerotolerant bacteria differs from its proportion among *Enterobacterales *alone. Further research is required to determine the most appropriate denominator for relative abundance in this context, considering that the main aerotolerant bacteria found in the gastrointestinal tract of ICU patients are *Enterobacterales *when SDD was not used [51]. While this may not capture the full microbial context, our study aimed to evaluate a pragmatic and clinically applicable approach to this tool. Further research is needed to explore the role of RAC in predicting ESBL-E-related infections and to develop effective predictive tools for AMS in this high-risk population.

## Conclusion

In conclusion, the RAC (using the total aerotolerant bacterial count as the denominator) of ESBL-E rectal carriage was not associated with confirmed ESBL-E-related VAP. However, rectal colonization with ESBL non-*Escherichia coli* strains was associated with confirmed ESBL-E-related VAP. Further research is needed to assess the utility of RAC and to explore effective strategies for improving AMS in ESBL-E carriers with suspected VAP.

## Supplementary Information

Supplementary Material 1: eFigure 1. Evolution of relative faecal abundance of ESBL-E in consecutive rectal swabs among the 83 carriers with confirmed ventilator-associated pneumonia. Categories of ESBL-E faecal relative abundance were defined as low, mediumor High. ESBL-E, extended-spectrum β-lactamase-producing *Enterobacterales.*

Supplementary Material 2.

Supplementary Material 3.

Supplementary Material 4.

Supplementary Material 5.

## Data Availability

The datasets used and/or analysed during the current study are available from the corresponding author on reasonable request.

## Abbreviations

AMS: Antimicrobial stewardship
ARDS: Acute respiratory distress syndrome
CFU: Colony-forming units
CRB: Carbapenem-resistant bacteria
ESBL-E: Extended-spectrum β-lactamase-producing *Enterobacterales*
GNB: Gram negative bacteria
ICU: Intensive care unit
MDR: Multidrug-resistant
MV: Mechanical ventilation
RAC: Relative abundance of ESBL-E rectal carriage
SAPS: Simplified acute physiology score
SDD: Selective digestive decontamination
SOFA: Sequential organ failure assessment
TSA: Tryptic soy agar
UTI: Urinary tract infections
VAP: Ventilator-associated pneumonia
