# Supplementary material for: Relative faecal abundance to predict extended-spectrum β-lactamase-producing Enterobacterales related ventilator‑associated pneumonia
Source: Ann Intensive Care. 2025 Mar 20;15:34. doi: 10.1186/s13613-025-01456-w (PMC11925845; doi:10.1186/s13613-025-01456-w)
Supplement: Supplementary file 1 — Supplementary Material 1: eFigure 1. Evolution of relative faecal abundance of ESBL-E in consecutive rectal swabs among the 83 carriers with confirmed ventilator-associated pneumonia. Categories of ESBL-E faecal relative abundance were defined as low, mediumor High. ESBL-E, extended-spectrum β-lactamase-producing Enterobacterales. [file 13613_2025_1456_MOESM1_ESM.pptx]

## Slide 1
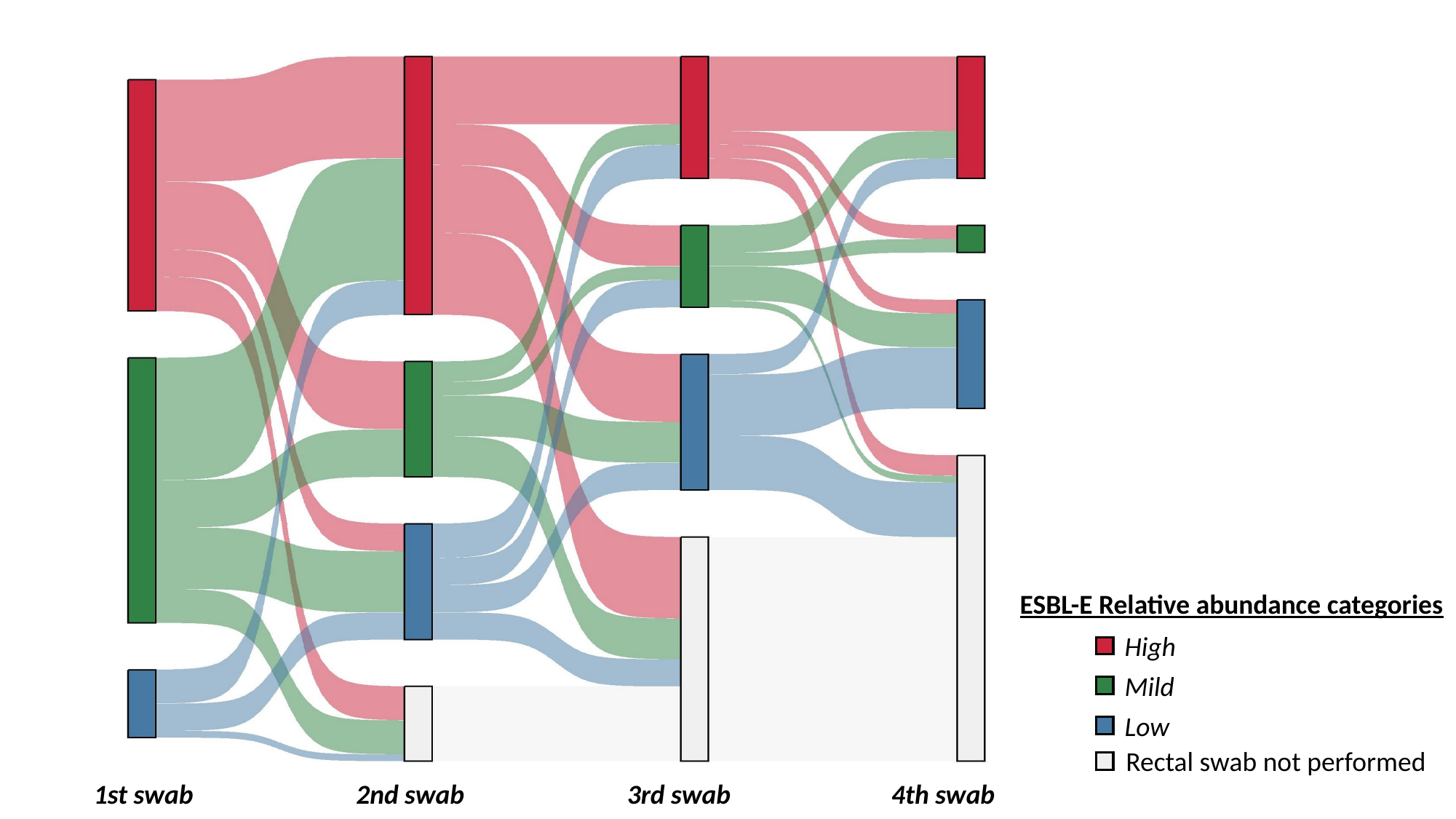

ESBL-E Relative abundance categories
High
Mild
Low
Rectal swab not performed
4th swab
1st swab
2nd swab
3rd swab
