# Supplementary material for: Relative faecal abundance to predict extended-spectrum β-lactamase-producing Enterobacterales related ventilator‑associated pneumonia
Source: Ann Intensive Care. 2025 Mar 20;15:34. doi: 10.1186/s13613-025-01456-w (PMC11925845; doi:10.1186/s13613-025-01456-w)
Supplement: Supplementary file 2 — Supplementary Material 2. [file 13613_2025_1456_MOESM2_ESM.docx]

| **eTable 1. Curative antibiotic therapy after antimicrobial susceptibility testing results** | | |
| --- | --- | --- |
| **Variable** | **ESBL-E related VAP group, n=62** | **Non ESBL-E related VAP, n=69** |
| Amoxicillin | 0 | 1 (1) |
| Amoxicillin-clavulanate | 0 | 4 (6) |
| Cloxacillin | 0 | 4 (6) |
| Piperacillin | 0 | 2 (3) |
| Cefotaxime | 0 | 6 (9) |
| Ceftazidime | 0 | 7 (10) |
| Cefepime | 0 | 18 (26) |
| Imipeneme | 10 (16) | 1 (1) |
| Meropeneme | 43 (69) | 6 (9) |
| Ceftazidime avibactam | 7 (11) | 0 |
| Ceftolozane-tazobactam | 0 | 1 (1) |
| Imipenem-relebactam | 0 | 1 (1) |
| Azactam | 3 (5) | 0 |
| Cefiderocol | 1 (2) | 0 |
| Ciprofloxacin | 3 (5) | 3 (4) |
| Levofloxacin | 2 (3) | 13 (19) |
| Polymyxin | 2 (3) | 0 |
| Fosfomycin | 2 (3) | 0 |
| Sulfamethoxazole-trimethoprim | 3 (5) | 2 (3) |
| Tigecycline | 1 (2) | 0 |
| Vancomycin | 1 (2) | 7 (10) |
| Linezolid | 0 | 2 (3) |
| Abbreviations: ESBL-E, extended-spectrum β-lactamase-producing *Enterobacterales* | | |
