# Supplementary material for: Relative faecal abundance to predict extended-spectrum β-lactamase-producing Enterobacterales related ventilator‑associated pneumonia
Source: Ann Intensive Care. 2025 Mar 20;15:34. doi: 10.1186/s13613-025-01456-w (PMC11925845; doi:10.1186/s13613-025-01456-w)
Supplement: Supplementary file 3 — Supplementary Material 3. [file 13613_2025_1456_MOESM3_ESM.docx]

| **eTable 2. Abundance of ESBL-E colonisation on last rectal screening available on the day of the VAP according to ESBL-E related VAP status** | | | |
| --- | --- | --- | --- |
| **Variable** | **ESBL-E related VAP, n=62** | **Non ESBL-E related VAP, n=69** | **p** |
| Relative abundance of ESBL-E colonisation on last rectal screening available on the day of the VAP | | | |
| Whole cohort (*N*=131) | | | 0.20 |
| 0 | 5 (8) | 4 (6) |  |
| 0.01% | 6 (10) | 1 (1) |  |
| 0.1% | 2 (3) | 4 (6) |  |
| 1% | 6 (10) | 8 (12) |  |
| 10% | 16 (26) | 25 (36) |  |
| 100% | 27 (43) | 27 (39) |  |
| - ESBL-E rectal carriage with *Escherichia coli* alone (*N*=65) | | | 0.10 |
| 0 | 0 | 0 |  |
| 0.01% | 1 (4) | 1 (2) |  |
| 0.1% | 0 | 2 (5) |  |
| 1% | 1 (4) | 5 (12) |  |
| 10% | 7 (29) | 15 (37) |  |
| 100% | 15 (63) | 18 (44) |  |
| - ESBL-E rectal carriage with others ESBL-E than *Escherichia coli* (*N*=66) | | | 0.22 |
| 0 | 5 (13) | 4 (14) |  |
| 0.01% | 5 (13) | 0 |  |
| 0.1% | 2 (5) | 2 (7) |  |
| 1% | 5 (13) | 3 (11) |  |
| 10% | 9 (24) | 10 (36) |  |
| 100% | 12 (32) | 9 (32) |  |
| Duration between screening and VAP ≤ 7 days and bacterial load on rectal swab > 10^4^ CFU/mL (*N*=85) | | | 0.10 |
| 0 | 4 (11) | 2 (4) |  |
| 0.01% | 3 (8) | 1 (2) |  |
| 0.1% | 2 (5) | 3 (6) |  |
| 1% | 2 (5) | 4 (8) |  |
| 10% | 11 (30) | 18 (38) |  |
| 100% | 15 (41) | 20 (42) |  |
| First episode of VAP (*N*=83) | | | 0.45 |
| 0 | 2 (6) | 3 (6) |  |
| 0.01% | 1 (3) | 1 (2) |  |
| 0.1% | 2 (6) | 4 (8) |  |
| 1% | 3 (9) | 5 (10) |  |
| 10% | 9 (28) | 17 (33) |  |
| 100% | 15 (47) | 21 (41) |  |
| Absolute abundance of ESBL-E colonisation on last rectal screening available on the day of the VAP (CFU/mL) | | | |
| Whole cohort (*N*=131) | | | 0.48 |
| 0 | 5 (8) | 4 (6) |  |
| 10 | 13 (21) | 6 (9) |  |
| 10² | 7 (11) | 7 (10) |  |
| 10^3^ | 9 (15) | 8 (11) |  |
| 10^4^ | 13 (21) | 29 (42) |  |
| 10^5^ | 15 (24) | 15 (22) |  |
| Results are *N* (%)  Abbreviations: CFU, colony forming unit; ESBL-E, extended-spectrum β-lactamase-producing *Enterobacterales*; VAP, ventilator associated pneumonia  Two-tailed *p*-values come from unadjusted comparisons using Cochran–armitage trend test. No adjustment for multiple comparisons was performed. | | | |
