# Supplementary material for: Relative faecal abundance to predict extended-spectrum β-lactamase-producing Enterobacterales related ventilator‑associated pneumonia
Source: Ann Intensive Care. 2025 Mar 20;15:34. doi: 10.1186/s13613-025-01456-w (PMC11925845; doi:10.1186/s13613-025-01456-w)
Supplement: Supplementary file 4 — Supplementary Material 4. [file 13613_2025_1456_MOESM4_ESM.docx]

| **eTable 3. Predictive value of Relative abundance of ESBL-E rectal colonisation to diagnose ESBL-E related VAP** | | | | |
| --- | --- | --- | --- | --- |
| **Relative abundance of ESBL-E rectal colonisation (semi-quantitative)** | **Sensitivity %**  **(95% CI)** | **Specificity %**  **(95% CI)** | **PPV %**  **(95% CI)** | **NPV %**  **(95% CI)** |
| **Global cohort (*N*=131)** | | | | |
| ≥0.1% (i.e., 0% / 0.01% vs. 0.1% / 1% / 10% / 100%) | 82 [70-91] | 7 [2-16] | 44 [41-48] | 31 [14-55] |
| ≥1% (i.e., 0% / 0.01% / 0.1% vs. 1% / 10% / 100%) | 79 [67-88] | 13 [6-23] | 45 [41-49] | 41 [24-60] |
| ≥10% (i.e., 0% / 0.01% / 0.1% / 1% vs. 10% / 100%) | 69 [56-80] | 25 [15-36] | 45 [40-51] | 47 [34-61] |
| ≥100% (i.e., 0% / 0.01% / 0.1% / 1% / 10% vs. 100%) | 44 [31-57] | 61 [48-72] | 50 [40-60] | 55 [47-62] |
| **Rectal colonisation with ESBL-producing *Escherichia coli* alone (*N*=65)** | | | | |
| ≥0.1% (i.e., 0% / 0.01% vs. 0.1% / 1% / 10% / 100%) | 95 [79-100] | 2 [0-13] | 37 [34-39] | 50 [6-94] |
| ≥1% (i.e., 0% / 0.01% / 0.1% vs. 1% / 10% / 100%) | 96 [79-100] | 7 [2-20] | 38 [35-41] | 75 [25-96] |
| ≥10% (i.e., 0% / 0.01% / 0.1% / 1% vs. 10% / 100%) | 92 [73-99] | 20 [9-35] | 40 [35-45] | 80 [48-95] |
| ≥100% (i.e., 0% / 0.01% / 0.1% / 1% / 10% vs. 100%) | 62 [41-81] | 56 [40-72] | 45 [34-57] | 72 [59-82] |
| **Rectal colonisation with ESBL-producing non-*Escherichia coli* ESBL (*N*=66)** | | | | |
| ≥0.1% (i.e., 0% / 0.01% vs. 0.1% / 1% / 10% / 100%) | 74 [57-87] | 14 [4-33] | 54 [48-60] | 29 [12-53] |
| ≥1% (i.e., 0% / 0.01% / 0.1% vs. 1% / 10% / 100%) | 68 [51-82] | 21 [8-41] | 54 [47-61] | 33 [17-54] |
| ≥10% (i.e., 0% / 0.01% / 0.1% / 1% vs. 10% / 100%) | 55 [38-71] | 32 [16-52] | 52 [43-62] | 35 [22-50] |
| ≥100% (i.e., 0% / 0.01% / 0.1% / 1% / 10% vs. 100%) | 32 [17-49] | 68 [48-84] | 57 [40-73] | 42 [34-51] |
| Abbreviations: ESBL-E, extended-spectrum β-lactamase-producing *Enterobacterales*; NPV, negative predictive value; PPV, positive predictive value; VAP, ventilator associated pneumonia | | | | |
