# Supplementary material for: Relative faecal abundance to predict extended-spectrum β-lactamase-producing Enterobacterales related ventilator‑associated pneumonia
Source: Ann Intensive Care. 2025 Mar 20;15:34. doi: 10.1186/s13613-025-01456-w (PMC11925845; doi:10.1186/s13613-025-01456-w)
Supplement: Supplementary file 5 — Supplementary Material 5. [file 13613_2025_1456_MOESM5_ESM.docx]

| **eTable 4**. Summary of studies investigating the relationship between ESBL-E abundance and the occurrence of ESBL-E related infections | | | | | | | | |
| --- | --- | --- | --- | --- | --- | --- | --- | --- |
| Author, year, reference | Country | Type of study | Patients | Sample | ESBL-E abundance | ESBL-E counts calculation | Denominator | Effect |
| Ruppé, 2013 (1) | Moldova, Turkey, Romania and Greece | Cross-sectional study | 31 female patients with *E. coli* UTI who were not exposed to antibiotics when the stool was passed  (outpatients) | Stool sample | Relative | Plating serial dilutions on Drigalski agar. CFU were counted in decimal logarithms at the dilution in which 1 to 100 CFU grew | Total number of EB | Association: The mean ESBL-RA was 18-fold higher in women with ESBL *E. coli* UTI than in those with non-ESBL *E. coli* UTI (10.0% [0.54%-100%] versus 0.56% [0.15%-2.1%]; P < 0.05). An ESBL-RA of <0.1% was 100% predictive of a non-ESBL E. coli UTI. |
| Woerther, 2015 (2) | France | Monocentric retrospective study | 104 patients with haematological malignancies (AML or HSCT) with EB related-BSI  (hospital ward) | Stool sample | Absolute | Plating on on Drigalski agar. ESBL-E level carriage was quoted as (0), (+), (++)or(+++) when 0, 1 to 9, 10 to 99 or 100 and more colonies were grown on the plates | n.a | Association: the patients with the highest level of ESBL-E rectal carriage were  those with the highest risk of bacteraemia due to ESBL-E (P<0.001) |
| Andremont, 2020 (3) | France | Monocentric prospective cohort | 107 ESBL-E carriers with GNB-related VAP  (ICU) | Rectal swab | Absolute | Streaking of the liquid of the rectal swab using the four-quadrant streak plate method. Densities of colonization were defined by visual semi-quantification (number of CFU and number of quadrants with colonies) | n.a | Association: Multivariable analysis showed that ESBL-E VAP was associated with significantly higher ESBL-E density in rectal swabs. |
| Pilmis, 2021 (4) | France | Monocentric prospective cohort | 24 ESBL-E rectal carriers treated for a microbiologically documented infection.  (ICU), 1^st^ source of infection was UTI (41.6%) | Stool sample | Relative | Plating serial dilutions on Drigalski agar. CFU were counted in decimal logarithms at the dilution in which 1 to 100 CFU grew | Total number of EB | Association: The mean ESBL-RA was more than 10-fold higher for the patients ESBL-E related infection than for the discordant patients (59.1% vs 4.9%; P < 0.001). |
| Abbreviations: CFU, colony-forming unit; EB, *Enterobacterales*; ESBL-E, extended-spectrum β-lactamase-producing *Enterobacterales*; ICU, intensive care unit; RA, relative abundance; VAP, ventilator associated pneumonia; UTI, urinary tract infections | | | | | | | | |

**REFERENCE**

1. Ruppé E, Lixandru B, Cojocaru R, Büke C, Paramythiotou E, Angebault C, et al. Relative fecal abundance of extended-spectrum-β-lactamase-producing Escherichia coli strains and their occurrence in urinary tract infections in women. Antimicrob Agents Chemother. 2013 Sep;57(9):4512–7.

2. Woerther PL, Micol JB, Angebault C, Pasquier F, Pilorge S, Bourhis JH, et al. Monitoring antibiotic-resistant enterobacteria faecal levels is helpful in predicting antibiotic susceptibility of bacteraemia isolates in patients with haematological malignancies. J Med Microbiol. 2015 Jul;64(7):676–81.

3. Andremont O, Armand-Lefevre L, Dupuis C, de Montmollin E, Ruckly S, Lucet JC, et al. Semi-quantitative cultures of throat and rectal swabs are efficient tests to predict ESBL-Enterobacterales ventilator-associated pneumonia in mechanically ventilated ESBL carriers. Intensive Care Med. 2020 Jun;46(6):1232–42.

4. Pilmis B, Mizrahi A, Péan de Ponfilly G, Philippart F, Bruel C, Zahar JR, et al. Relative faecal abundance of extended-spectrum β-lactamase-producing Enterobacterales and its impact on infections among intensive care unit patients: a pilot study. J Hosp Infect. 2021 Jun;112:92–5.
